# Liquid-phase exfoliated graphene: functionalization, characterization, and applications

**DOI:** 10.3762/bjnano.5.242

**Published:** 2014-12-04

**Authors:** Mildred Quintana, Jesús Iván Tapia, Maurizio Prato

**Affiliations:** 1 Instituto de Física, Universidad Autónoma de San Luis Potosí, Manuel Nava 6, Zona Universitaria 78290, San Luis Potosí, SLP, Mexico; 2 Center of Excellence for Nanostructured Materials (CENMAT), INSTM UdR di Trieste, Dipartimento di Scienze Chimiche e Farmaceutiche, University of Trieste, Piazzale Europa 1, 34127 Trieste, Italy

**Keywords:** applications, dispersions, graphene, organic functionalization, ultrasonication

## Abstract

The development of chemical strategies to render graphene viable for incorporation into devices is a great challenge. A promising approach is the production of stable graphene dispersions from the exfoliation of graphite in water and organic solvents. The challenges involve the production of a large quantity of graphene sheets with tailored distribution in thickness, size, and shape. In this review, we present some of the recent efforts towards the controlled production of graphene in dispersions. We also describe some of the chemical protocols that have provided insight into the vast organic chemistry of the single atomic plane of graphite. Controlled chemical reactions applied to graphene are expected to significantly improve the design of hierarchical, functional platforms, driving the inclusion of graphene into advanced functional materials forward.

## Review

### Introduction

Various methodologies for the production of graphene and chemically modified graphene have been described during the last years [1]. Among them, micromechanical exfoliation [2] and metal supported growth [3] produced the best layers in terms of electrical and structural quality. Unfortunately, these strategies are time consuming, expensive or produce low material yield. It is crucial to push these methodologies forward towards the large-scale and low-cost production of graphene for the development of applications. Recently, using chemical vapour deposition (CVD) techniques (as reported in [4]), it was possible to produce transferable, large graphene sheets that were included in transparent conductive films for mobile phones [5]. However, for the development of composite materials in large quantities, good quality graphene dispersions must be produced. In this direction, wet chemistry methodologies are proficient for the mass fabrication of graphene, since the starting material is usually graphite, an inexpensive substance [6].

To exfoliate graphite by wet chemistry techniques, it is necessary to decrease the π–π staking interactions between the graphene layers. In achieving this, the sp^2^ lattice is partially disrupted into layers containing sp^2^–sp^3^ carbon atoms. The most drastic example is probably graphene oxide (GO), where the extreme oxidation of the carbon lattice causes a detriment in the electrical and mechanical properties of the sheets [7]. As consequence, even today, chemically-produced graphene is suitable for the development of low performance applications. In order to produce graphene dispersions within satisfactory optical and electrical property specifications, experimental techniques must be improved. More research must be performed to better understand and manipulate: the mechanochemical forces required for weakening the van der Waals cohesive forces in graphite, the organic chemistry of the two-dimensional graphene layers and the colloidal mechanisms that guide stabilization of graphene sheets possessing diverse physical and chemical features such as charge, size and shape.

To date, the exfoliation of graphite in dispersions has been obtained by a number of mechanochemical methods. Each one of these strategies presents advantages and disadvantages depending on the type of application. In this contribution, we summarize some of the chemical procedures used to obtain graphene dispersions for various applications. Special emphasis is placed on the liquid-phase exfoliation of graphite by ultrasonic techniques, since this is a straightforward process for the production of clean graphene layers ready for organic functionalization. Importantly, the exfoliation of graphite by ultrasonication is a very versatile technique that has already demonstrated its effectiveness in dispersing graphene in solvents with different physical and chemical properties. This flexibility allows the incorporation of additives such as surfactants [8], antioxidants [9], and polymers [10] during the ultrasonication process, while increasing the affinity for the solvent, the quality of the resulting graphene layers, or their functionality. Finally, we describe recent efforts in the chemical design of tailored graphene nanohybrids using graphene dispersions as the starting material.

### Discussion

#### Graphene in dispersions

As previously mentioned, a number of chemical strategies can be followed for the production of graphene in dispersions. Until now, the most widely used methodology has involved the production of GO [11]. In this approach, strong oxidizing agents cause exfoliation of graphite by altering the chemical structure of the 2D graphene layer, introducing oxygen moieties that constrain π–π stabilization. Oxidation of the carbon lattice creates organic functional groups such as epoxides, alcohols, ketones, carbonyls, and carboxylic groups [12]. As result, GO presents completely different physical and chemical properties from that of graphene. The presence of oxidized carbon species modifies the conductive properties of GO from that of a semimetal to an insulating material, while its chemical stability is considerably reduced.

As an alternative, charged species can be used to induce the exfoliation of graphite. For example, the addition of electrons from alkali metal intercalation compounds resulted in the spontaneous exfoliation of graphite in water or polar solvents. This procedure yields stable solutions of negatively charged graphene sheets [13].

As charged, intercalating species, ionic liquids are considered a green alternative. The negligible vapour pressure, thermal stability, wide electrochemical potential window, good conductivity, recyclability, and the high dielectric constant of ionic liquids induce the exfoliation of graphite by weakening the π–π stacking interactions. Indeed, the use of ionic liquids is considered a highly versatile and industrially scalable method for the preparation of graphene nanomaterials [14].

Some neutral molecules are also able to exfoliate graphite by producing polarized π-systems [15]. For example, we recently reported the production of graphene layers by the mechanochemical activation of graphite with melanine (2,4,6-triamine-1,3,5-triazine) under solid conditions through a ball milling process [16]. The melamine-coated graphene layers are easily dispersed in polar solvents. Later, following a similar methodology, the stabilization of a high concentration of graphene sheets in different solvents was achieved by using diverse aminotriazine molecules [17].

The methodologies used for the exfoliation of graphite described thus far might pose a challenge to the further modification of graphene layers by covalent organic reactions. The presence of additional chemical species could yield unexpected products. Thus, different approaches are followed for the production of clean graphene sheets. For example, graphene can be produced by supercritical solvent exfoliation of graphite. In this procedure, solvents reach or exceed their critical point, presenting outstanding wetting properties, low interfacial tension, low viscosity, and high diffusion coefficients. Under these conditions, graphite is exfoliated as high-quality graphene [18].

#### Ultrasonic techniques

**Figure 1:**
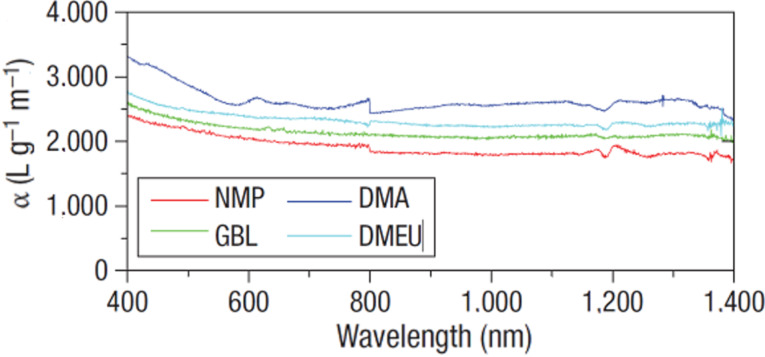
Absorption spectra of graphene layers dispersed in *N*-methylpyrrolidone (NMP), γ-butyrolactone (GBL), *N*,*N*-dimethylacetamide (DMA) and 1,3-dimethyl-2-imidazolidinone (DMEU) at concentrations from 2 to 8 µg∙mL^−1^. Reprinted with permission from [21], copyright 2008 Nature Publishing Group.

A versatile and simple strategy to exfoliate graphite in liquid phase is based on the use of ultrasonic wave treatment. Ultrasound techniques have important applications in a wide range of materials synthesis strategies [19]. The physical and chemical phenomena associated with ultrasonic waves are cavitation and nebulization. Cavitation induces extreme conditions by collapsing air bubbles which initiates chemical reactions, while nebulization furthers the reaction within the heated droplets. These processes induce the production of highly reactive chemical species including peroxides and radicals formed from the sonochemical reaction of the solvent in air [20]. The combined mechanochemical effects of ultrasonication prompt the exfoliation of graphite in organic solvents, surfactant solutions, or mixed organic solvents. After the ultrasonication process, graphene dispersions are typically centrifuged in order to eliminate graphite microcrystals. From the obtained, stable dispersions, it is possible to calculate the concentration of the graphene layers by UV–vis absorption, as shown in Figure 1 [21]. This protocol allows the quantification of the graphene sheets in the dispersion, enabling further organic functionalization by stoichiometric reactions. This approach is suitable for the tailored design of materials with applications in many different research fields [22].

Ultrasonic processes are successful at exfoliating graphite only if the net energetic cost of the process is very small. The enthalpy of mixing depends on the affinity between graphene layers and solvent molecules. Then, in order to obtain high yields of exfoliated graphite, the surface energy of the solvent must compete with the surface energy of graphite (≈70–80 mJ∙m^−2^). This relatively high surface energy results in solvents with high boiling points and high surface tensions, for example, *N*,*N*-dimethylformamide (DMF) and *N*-methyl-1,2-pyrrolidone (NMP), which are well-known as good dispersing solvents for carbon nanostructures such as fullerenes and carbon nanotubes (CNTs) [23–25]. Using these solvents, it is possible to exfoliate graphite, resulting in defect-free graphene layers of high concentration. One limitation of this methodology is its inability to completely eliminate the absorbed solvent from the graphene surface. The strong molecular interactions between graphene layers and DMF or NMP molecules, in addition to the fact that both solvents have a high boiling point and high surface tension, make their complete evaporation or removal very difficult. The presence of these residual molecules modifies the physical properties of graphene, which is detrimental to their electrical performance.

As an alternative, it is possible to prepare graphene dispersions in chloroform, acetone, or isopropanol. These are all volatile solvents that have generally lower surface tensions, and are therefore more easily evaporated. Unfortunately, in order to achieve graphene layers of reasonable concentrations in volatile solvents, it is necessary to increase the ultrasonication time, which increases the oxidative processes that cut the graphene sheets into smaller layers, resulting in a higher content of oxidized carbon atoms [26].

The exfoliation of graphite in water is also possible by adding surfactants as stabilizing agents. For example, graphite and sodium cholate were ultrasonicated in water for long periods up to 400 h [27]. This process easily produces stable dispersions of high-quality, free-standing graphene films. In order to obtain more information related to the interaction between graphene sheets and surfactants, graphene was stabilized in water dispersions using twelve different surfactants [28]. The authors found that ionic surfactants stabilize graphene sheets with a concentration that increases with the square of the zeta potential of the surfactant-coated sheets. This means that the concentration is proportional to the magnitude of the electrostatic potential barrier, which prevents graphene π–π stabilization. In contrast, for non-ionic surfactants, the dispersed graphene concentration increased linearly with the magnitude of the steric potential barrier.

#### Production of larger graphene sheets

**Figure 2:**
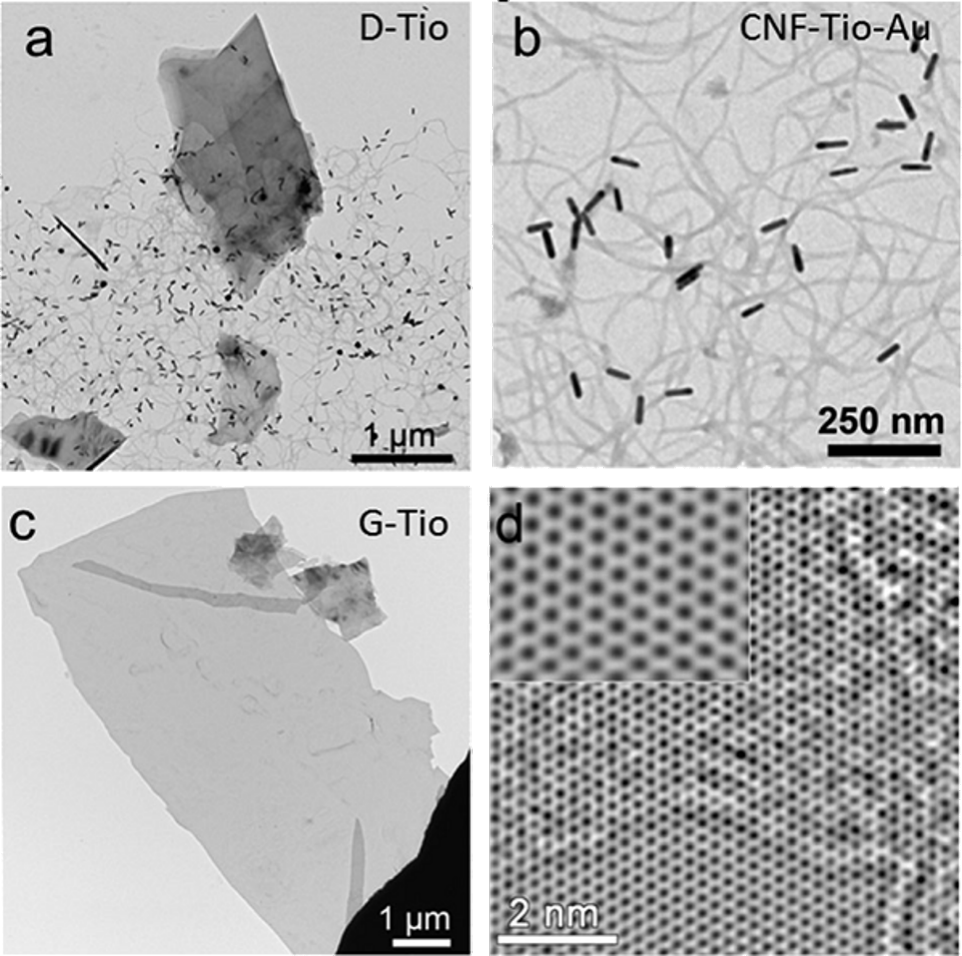
Carbon nanostructures produced with the addition of tiopronin. (a) TEM micrograph of a solution cast including graphene layers and carbon nanofibers. (b) Carbon nanofibers marked with Au Nanorods. (c) Representative TEM micrograph of a graphene sheet. (d) HR-TEM image of graphene. Reprinted with permission from [9], copyright 2012 The Royal Society of Chemistry.

As previously mentioned, ultrasonication is a highly energetic process that produces strongly oxidizing chemical species, such as radicals and peroxides. These radicals typically oxidize carbon atoms in graphene close to the defects and at the edges. Under prolonged periods of ultrasonication, the oxidation can cut graphene sheets into small pieces. A well-known strategy to mitigate the damage induced by radicals in different biological processes is through the addition of antioxidant. Following this approach, the ultrasonication of graphite upon addition of *N*-(2-mercaptopropionyl)glycine (tiopronin) was reported [9]. Tiopronin is an antioxidant molecule that traps electrons, radicals and peroxides. The ultrasonication of graphite in the presence of tiopronin produced larger graphene layers as compared to pure DMF. In this process, carbon nanofibers (CNFs) are formed revealing the occurrence of chemical reactions. During the ultrasonication process, graphene sheets were cut close to the edges, producing small fragments which later aggregate into CNFs. To verify the mechanism of CNF formation, gold nanoparticles (Au NPs) were introduced as contrast markers. Tiopronin and its fragments are well-known stabilizers for Au NPs. The analysis by transmission electron microscopy (TEM) showed Au NPs mostly resided on the nanofibers, thus supporting the proposed mechanism as shown in Figure 2. This methodology produces dispersions of larger graphene sheets of higher concentration than common ultrasonication in DMF. With this, it was demonstrated that the use of antioxidant molecules during ultrasonication treatments reduces the damage caused by radicals and other oxidizing chemical species, attacking mainly the edge carbon atoms.

#### Rolling and sealing graphene

**Figure 3:**
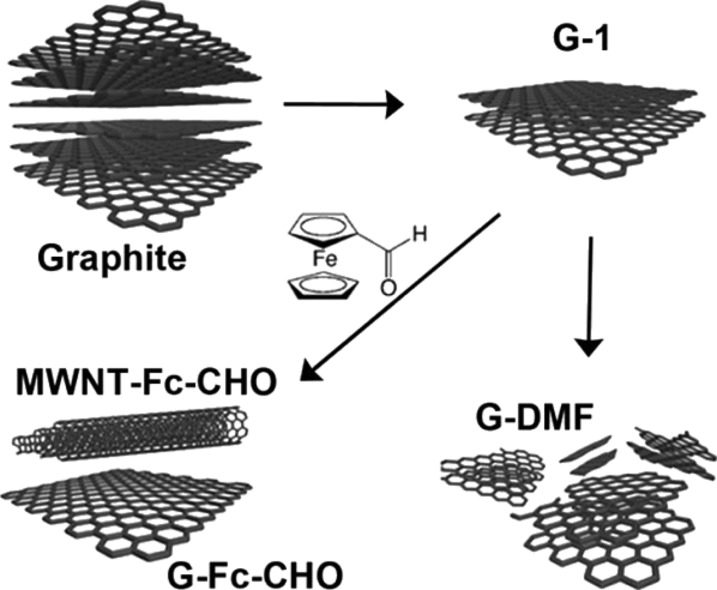
Ultrasound-assisted synthesis of MWNTs from graphite upon the addition of ferrocene aldehyde**.** Reprinted with permission from [30], copyright 2012 American Chemical Society.

An important potential application of exfoliated graphene is the tailored production of other carbon nanostructures such as fullerenes [29] and CNTs. To this effect, we have demonstrated the longstanding visualised strategy of rolling and sealing a graphene sheet [30]. This process is possible due to the ultrasonication of graphene upon addition of ferrocenecarboxaldehyde (Fc–CHO) in DMF, Figure 3. Fc–CHO is a reducing agent that prevents oxidation and radical reactions. Additionally, ferrocene derivatives are used in the synthesis of CNTs as carbon source and catalyst.

**Figure 4:**
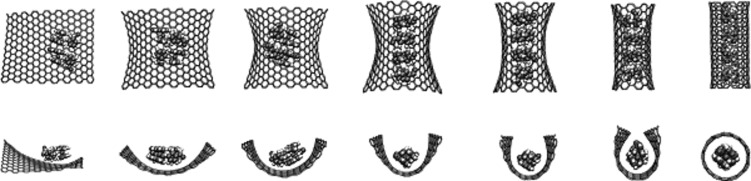
Slow-motion roll up of a graphene layer. Fc–CHO molecules template the rolling of the graphene sheet. Reprinted with permission from [31], copyright 2013 Wiley-VCH.

The proposed mechanism for the formation of the MWNTs from graphene is as follows [31]: (1) the production of graphene layers by ultrasonication of graphite in DMF, (2) the antioxidant effect of Fc–CHO which reduces the concentration of radical reactive species, reducing the cutting and damage of graphene layers, (3) the Fc–CHO adsorption at the edges of graphene layers inducing the rolling up of a sheet to form a nanoscroll (schematically indicated in Figure 4), (4) the trapping of Fc–CHO into the scroll, and (5) finally, Fc–CHO accepts and donates unpaired electrons to the graphene edges and converts the less stable scroll into a MWNT. This is an important step towards the controlled synthesis of carbon nanostructures. The interaction between graphene sheets and template molecules is expected to produce carbon nanostructures with well-defined properties such as diameter, chirality, shape and size.

#### Organic chemistry of graphene

From an organic chemistry perspective, graphene is a very exciting material as it can be functionalized on the two faces, at the edges and at the defects. Initially, graphene was expected to behave as a chemically inert surface, similar to graphite. However, it was experimentally and theoretically shown [32] that functional moieties attack graphene at both faces. This provides the changed energetics allowing the formation of chemical bonds that would be unstable if only one surface was exposed. This mechanism explains the exceptional reactivity of the graphene layer [33–34]. Unpaired electrons created at sites adjacent to the point of covalent bonding lead to a chain reaction from the initial point of attack [35]. For example, cycloaddition reactions onto graphene by means of first-principle calculations show a cooperative behaviour. The cycloaddition of benzynes were found to be energetically as strong as the attachment of hydrogen atoms, while for the 1,3-dipolar cycloaddition, the free energy of the reaction is slightly smaller [36]. Additionally, carbon atoms which localize at graphene edges are considered to be more reactive than carbon atoms at the bulk surface faces.

**Figure 5:**
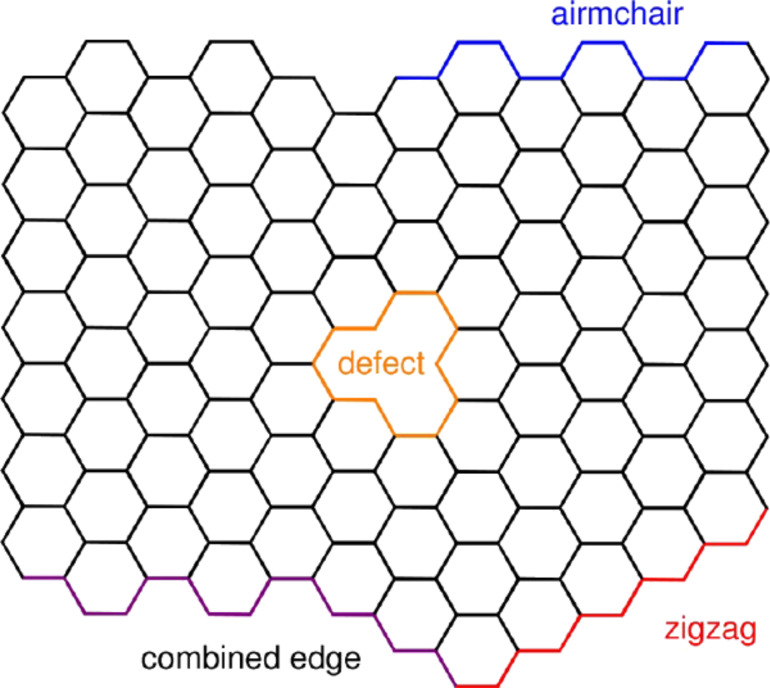
Schematic representation of reactive sites in graphene: surface faces, edges, and defects. Reprinted with permission from [22], copyright 2013 American Chemical Society.

There are two types or graphene edges: zigzag and armchair. Zigzag edges are considered more reactive as compared to the armchair ones (Figure 5). Defects are considered to be reactive sites as well. The chemical methods reported to date yield graphene with different types and amounts of defects, including damage in the carbon lattice, structural imperfections, adatoms, and solvent molecules randomly adsorbed.

Thus, reactions occur at both faces of the graphene layer and different types of edges show different chemical reactivity. Additionally, defects such as vacancies, adatoms or substitutional atoms might act as active chemical sites on the graphene layer [37]. These features make the organic chemistry of graphene very accessible, but only partially understood. Improvement in the chemical knowledge of the two dimensional material is expected to increase the tailored design of advanced materials.

The covalent organic functionalization of graphene is necessary for several purposes. For example: (1) to increase the dispersibility in common organic solvents and water and (2) to combine the properties of graphene with the properties of other functional materials such as nanoparticles, polymers or chromophores [38–39]. The appropriate design and construction of the graphene chemical surface are essential to reach the best material performance in the composite.

#### Visualizing graphene reactivity

Organic reactions might present different reactivity on the graphene surface when compared with other carbon nanostructures. The characterization of functionalized graphene layers typically requires the use of advanced analytic techniques. Graphene layers in dispersions are normally present at low concentrations and functional groups appear only sporadically. Thus, high-resolution techniques such as X-ray photoelectron spectroscopy (HR-XPS) and HR-TEM are very useful for the identification of functional groups.

A common organic reaction used for the functionalization of carbon nanostructures such as fullerenes, nanotubes, nano onions, nano horns and currently graphene, is the use of dienophiles as reactive species. One of the most successfully applied dienophiles to form covalent bonds with the hexagonal sp^2^ lattice of carbon nanostructures is the azomethine ylide [40]. This intermediate reacts through a 1,3-dipolar cycloaddition mechanism.

**Figure 6:**
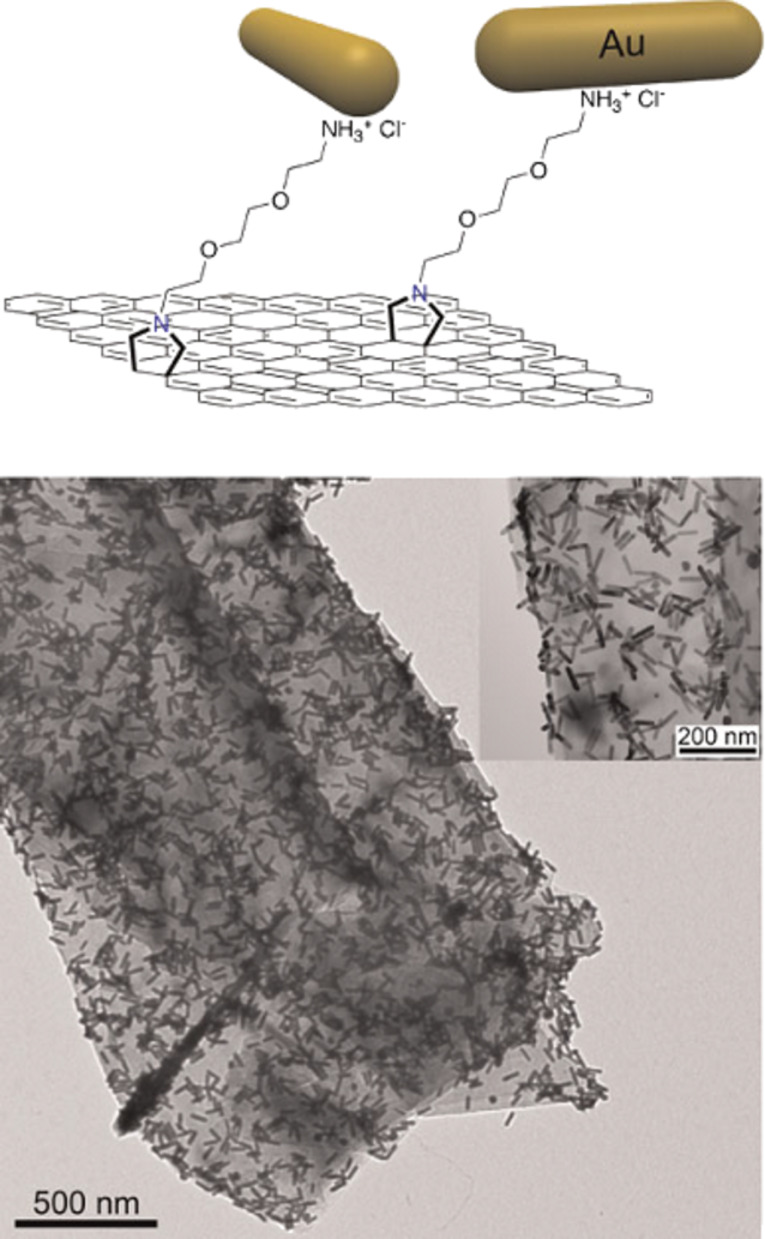
Graphene functionalization on the faces and edges by the 1,3-dipolar cycloaddition in DMF dispersions. Reproduced with permission from [41], copyright 2010 Wiley-VCH.

The 1,3-dipolar cycloaddition, performed on graphene by employing an aldehyde and an α-amino acid as precursors, attacks both graphene faces and edges. This reactivity was observed by introducing protonated terminal amino groups that selectively bind gold nanorods allowing the recognition of the reactive sites by low resolution TEM (Figure 6). The HR-XPS analysis allowed for the determination of the concentration of functional groups [41].

In order to acquire more knowledge related to the organic chemistry of exfoliated graphene by ultrasonication, carboxylic groups where introduced on the graphene sheets by applying the 1,3-dipolar cycloaddition. This product was compared with fresh exfoliated graphene by the derivatization of both products with PAMAN dendrons using an amide-bond condensation reaction [42]. Using Au NPs as markers, it was possible to visualize the reactive sites against each reaction.

**Figure 7:**
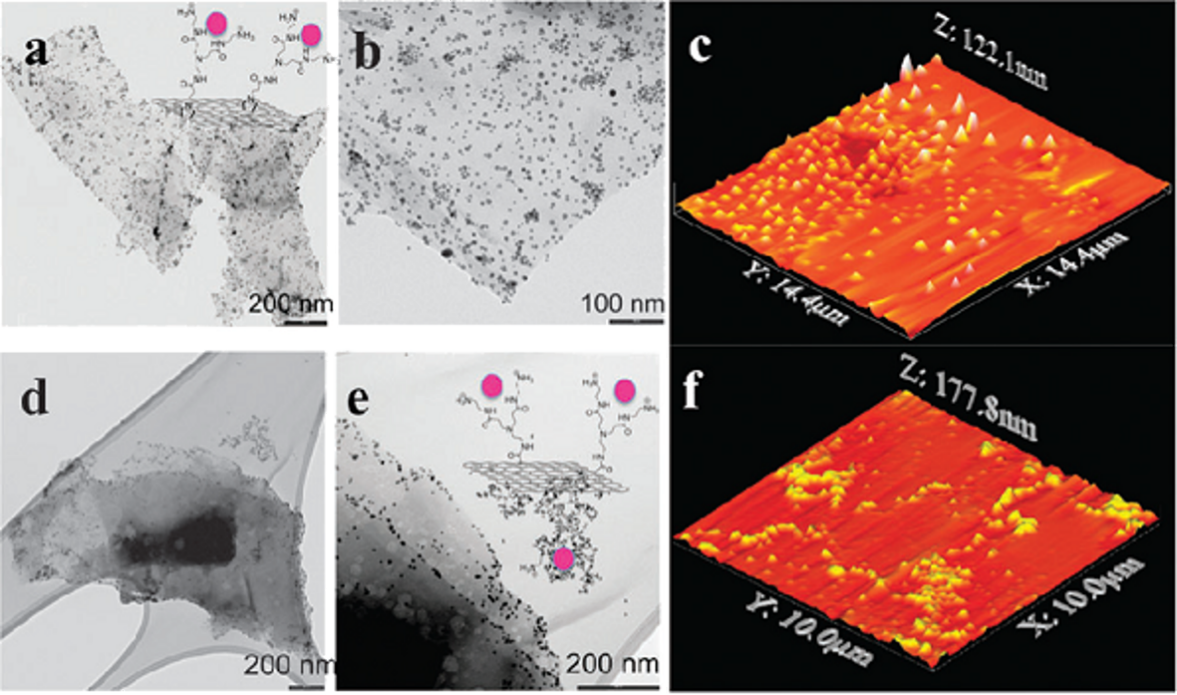
TEM and AFM micrographs after the addition of Au NPs for the identification of reactive sites against 1,3-dipolar cycloaddition (a–c) and amidation reaction on carboxylic groups created during the sonication process (d–f). Reproduced with permission from [42], copyright 2011 The Royal Society of Chemistry.

As expected, for the 1,3-dipolar cycloaddition reaction, the TEM micrographs showed Au NPs completely dispersed on graphene faces and edges. In contrast, for the amide-bond condensation reaction, Au NPs were mainly found on the graphene edges. These results were corroborated by HR-XPS analysis of the products. A higher concentration of functional groups was found in graphene functionalized by using the 1,3-dipolar cycloaddition. With this, we demonstrated that graphene sheets could be selectively functionalized on the borders or in the entire lattice (Figure 7).

These results corroborate the success of the production of graphene layers by ultrasonication techniques. Oxidized carbon atoms on graphene sheets are introduced mainly on the edges of the layers when compared with GO. Additionally, the presence of the adatoms is not revealed by HR-XPS or HR-TEM.

#### Graphene nanohybrids

**Figure 8:**
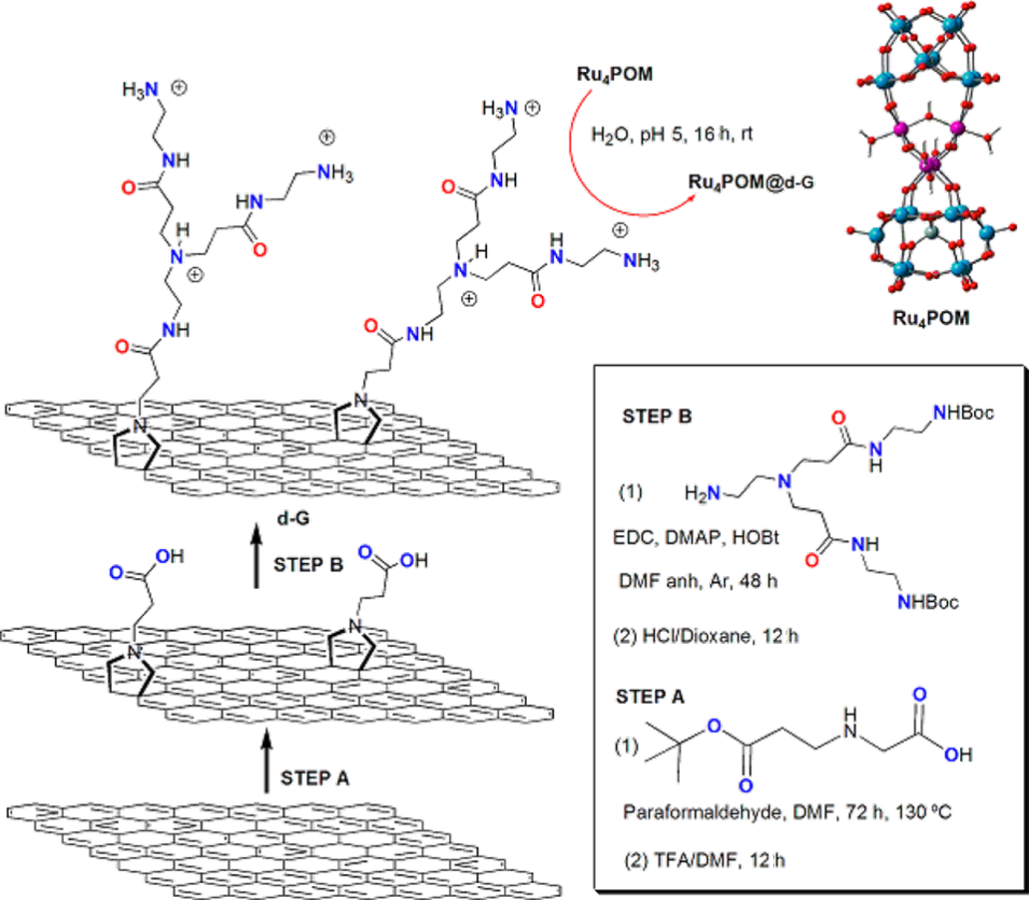
Synthesis of a graphene nano-platform supporting Ru_4_POM. Reproduced with permission from [43], copyright 2013 American Chemical Society.

As described above, organic chemical reactions can be chosen to attack the complete graphene sheets or mostly the edges. Strategies such as this enable the integration of the outstanding properties of graphene in different applications. For example, graphene–polyoxometalate hybrids are considered important materials for the development of artificial photosynthesis. The properties of graphene enhance the electrocatalytic performance of the catalytic polyoxometalate and minimize the applied overpotential, increasing the long-term robustness of the device [43]. Recently, we demonstrated the feasibility of mimicking the oxygen-evolving centre of natural PSII by using functionalized graphene by the 1,3-dipolar cycloaddition with positively charged dendrons. The charged moieties recognize the inorganic tetraruthenate (Ru_4_POM) anionic catalyst by electrostatic interactions, as illustrated in Figure 8. The device produced by deposition of the nanohybrid showed better performance when compared with similar substrates produced with functionalized CNTs. Our results support the importance of the rational chemical design of the carbon platform for the preservation of electron transport and accumulation processes associated with pristine graphene.

**Figure 9:**
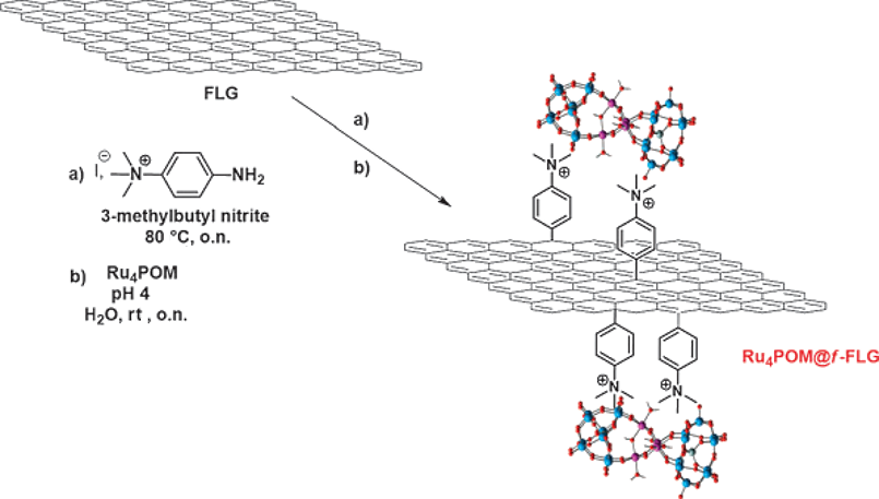
Synthesis of a polyoxometalate–graphene nanohybrid by using the diazonium-based reaction. Reproduced with permission from [45], copyright 2014 The Royal Society of Chemistry.

In order to introduce higher loadings of functional materials, stronger chemical reactions for graphene functionalization can be achieved by the attack of radical species. As reactive radical species, aryl diazonium salts are attractive and popular sources. These radicals covalently graft to carbon sp^2^ surfaces [44]. Using this radical reaction, graphene was covalently functionalized using a positively charged *N*,*N*,*N*-trimethylbenzenammonium, as illustrated in Figure 9 [45]. This strategy produces graphene layers with a substantial coverage of positive charges. Later, functional anions of Ru_4_POM in the charged functional moieties by complementary electrostatic interactions were introduced. Comparing the insertion of a shorter moiety by the diazonium arylation reaction with the positively charged dendrons attached by the 1,3-dipolar cycloaddition described before, we confirmed the interplay of electrostatic forces and π-electron clouds on graphene. The proximity of the Ru_4_POM to the graphene surface in the arylation reaction leads to a tight immobilization.

#### Imaging on graphene

**Figure 10:**
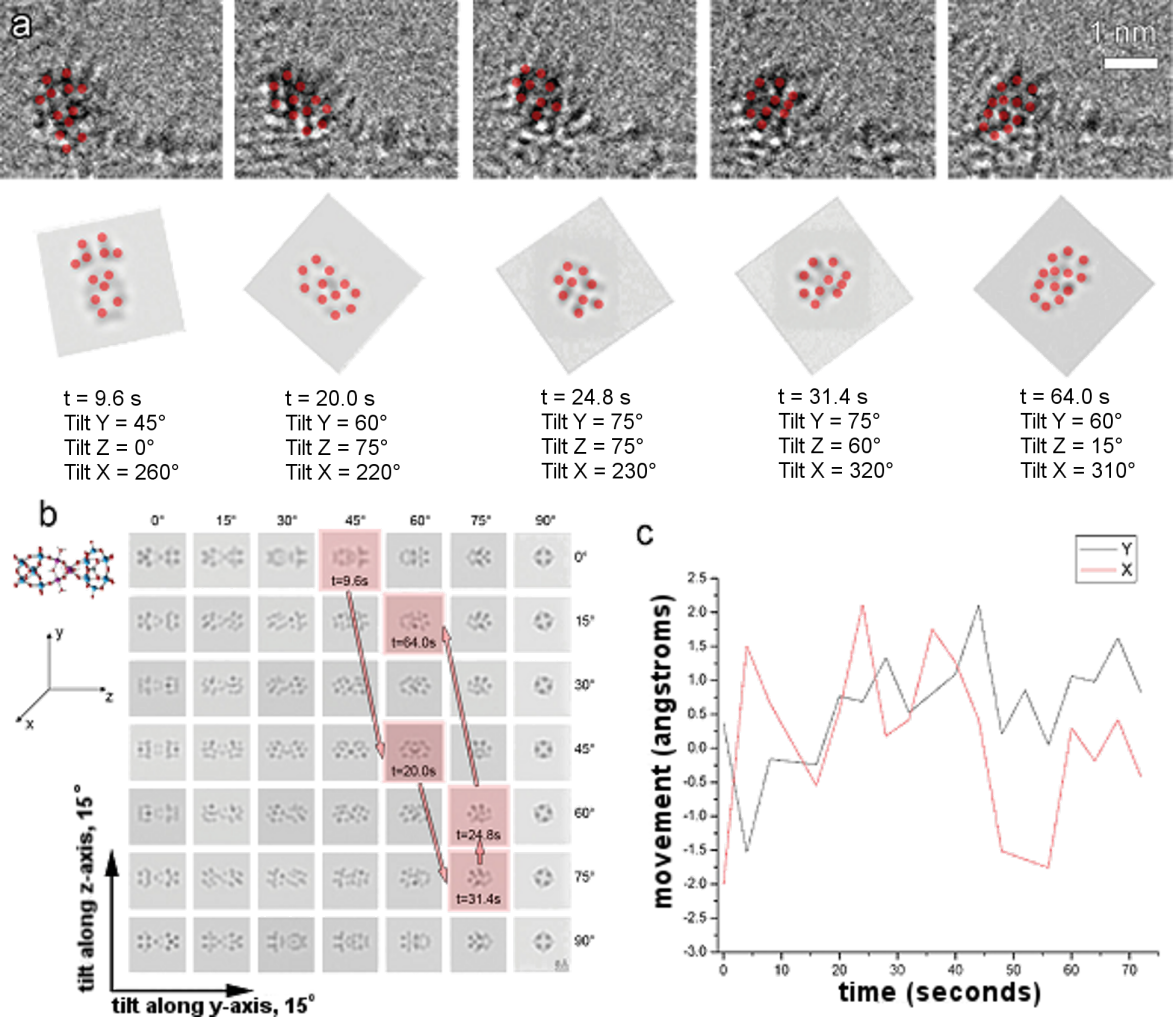
(a) Projections from a time sequence of dendron-functionalized Ru_4_POM together with their corresponding orientations. (b) A plot showing the orientation changes as highlighted by the red squares. The movement path is indicated by arrows. (c) A diagram showing 2D translation of Ru_4_POM with time. Reproduced with permission from [46], copyright 2013 Wiley-VCH.

Finally, the optical properties of graphene for the high contrast imaging of the two previously described graphene–polyoxometalate nanohybrids can be exploited. For this, Ru_4_POM molecules were identified on functionalized graphene surfaces by low voltage aberration-corrected TEM (AC-TEM) [46]. Following this, a time sequence analysis of the dynamical rotation of individual Ru_4_POM anions on functionalized graphene was performed. When comparing the Ru_4_POM anions accompanying short carbon moieties with those attached to dendrons with longer carbon chains, a higher degree of motion and frequency were observed for the latter, as shown in Figure 10. For both samples, the presence of the functional moiety constrains the lateral motion of the anion catalyst with no preference with respect to the graphene surface. Our results are exciting because this implies that the graphene morphology might influence the performance of functional materials. Thus, it is essential to carefully design the functional moieties to covalently attach to the graphene surface as well as the functionalization strategy.

## Conclusion

Substantial efforts have been performed for the mass scale-up and low-cost production of graphene. Thus far, wet chemical strategies have been demonstrated as the most efficient for the inclusion of graphene in low performance applications. To improve the physical properties of chemically produced graphene, it is necessary to gain better control of the experimental techniques by increasing the knowledge of the physical and chemical properties of both graphite and graphene. Likewise, it is critical to acquire detailed comprehension of the organic chemistry of the two dimensional material. The tailored chemical design of graphene platforms is essential for the development of new exciting functional materials.
